# Supplementary material for: Refinement strategies for Tangram for reliable single-cell to spatial mapping
Source: Bioinformatics. 2025 Jul 15;41(Suppl 1):i552–60. doi: 10.1093/bioinformatics/btaf194 (PMC12261478; doi:10.1093/bioinformatics/btaf194)
Supplement: btaf194_Supplementary_Data [file btaf194_supplementary_data.pdf]

Refinement Strategies for Tangram for Reliable Single-Cell to Spatial Mapping

Supplementary figures

List of supplementary figures

1 Hyperparameter tuning on real-world mouse cortex scRNA-seq and Visium data. 1
2 Benchmarking vanilla Tangram and refinement strategies. 2
3 Comparison of predicted gene expressions on real-world mouse cortex scRNA-seq and Visium data. 3
4 Comparison of cell mappings on real-world mouse cortex scRNA-seq and Visium data. 4
5 Comparison of cell mappings on simulated mouse hypothalamus scRNA-seq and spatial data. 5
6 Comparison of cell type mappings on real-world mouse cortex scRNA-seq and Visium data. 6
7 Comparison of cell type mappings on simulated mouse hypothalamus scRNA-seq and spatial data. 7
8 Cell and cell type mapping per cell type. 8
9 Runtimes analysis on real-world mouse cortex scRNA-seq and Visium data. 8

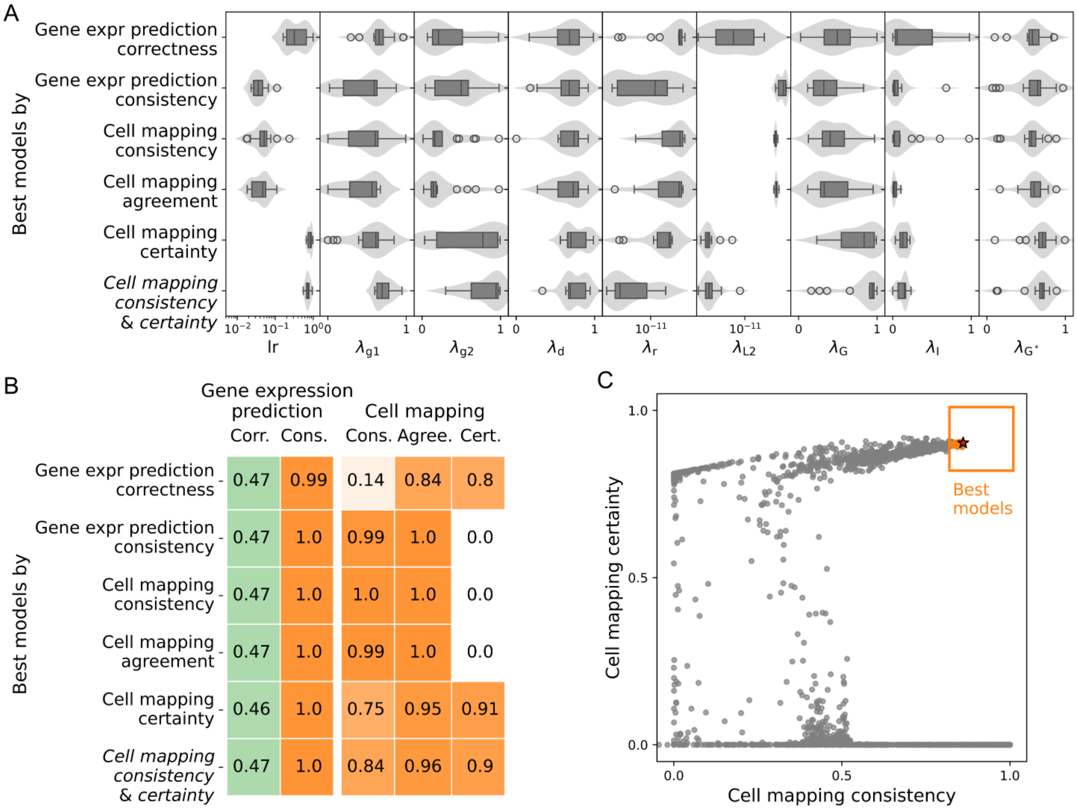

Fig. 1: **Hyperparameter tuning on real-world mouse cortex scRNA-seq and Visium data.** We refined the cell mapping alogrithm of Tangram by adding cell and gene selection, neighborhood incorporation, and regularization. We conducted 2,000 optimization trials using Optuna to fine-tune parameters using a Spapros geneset. (A) Hyperparameter value distributions and (B) benchmarking metrics for 20 best-performing models for different metric. (C) Discovery of the best-performing models regarding cell mapping consistency and certainty, with the orange box highlighting the region of best-performing models. The star marks our final selected model with the hyperparameter learning rate (lr) $= 0.72$, $\lambda_{g1} = 0.76$, $\lambda_{g2} = 0.99$, $\lambda_d = 0.89$, $\lambda_r = 2.95e^{-9}$, $\lambda_{L2} = 1.00e^{-18}$, $\lambda_G = 0.96$, $\lambda_I = 0.17$, and $\lambda_{G^*} = 0.71$.

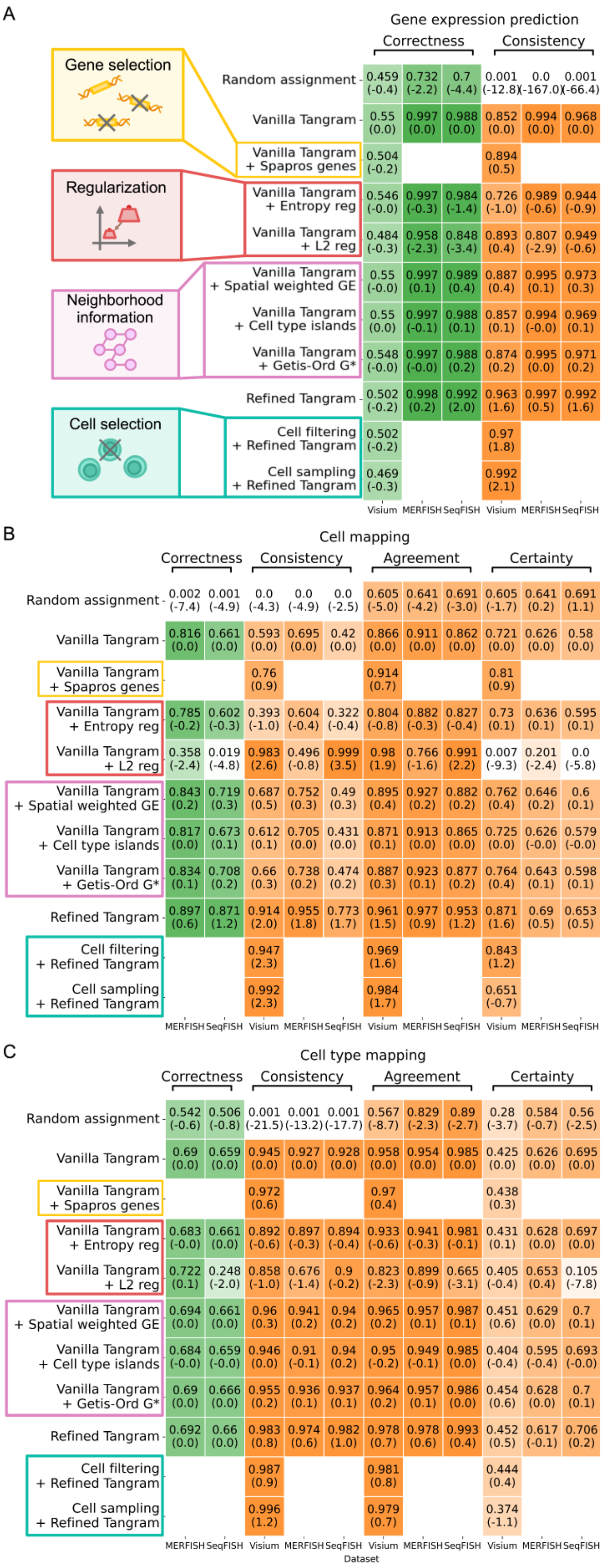

Fig. 2: **Benchmarking vanilla Tangram and refinement strategies.** We mapped cells of real-world and simulated dataset pairs using vanilla Tangram, different refinement strategies, and a refined version of Tangram that integrates all beneficial refinement strategies. We evaluated the performance of vanilla Tangram and the different refinement strategies by mapping cells from real-world and simulated dataset pairs. A random assignment of cells to spots is included as baseline. Metrics for ground truth comparisons are highlighted in green and metrics for repeated run comparisons in orange. Values in brackets are Cohen's d effect sizes in comparison to the vanilla Tangram performance.

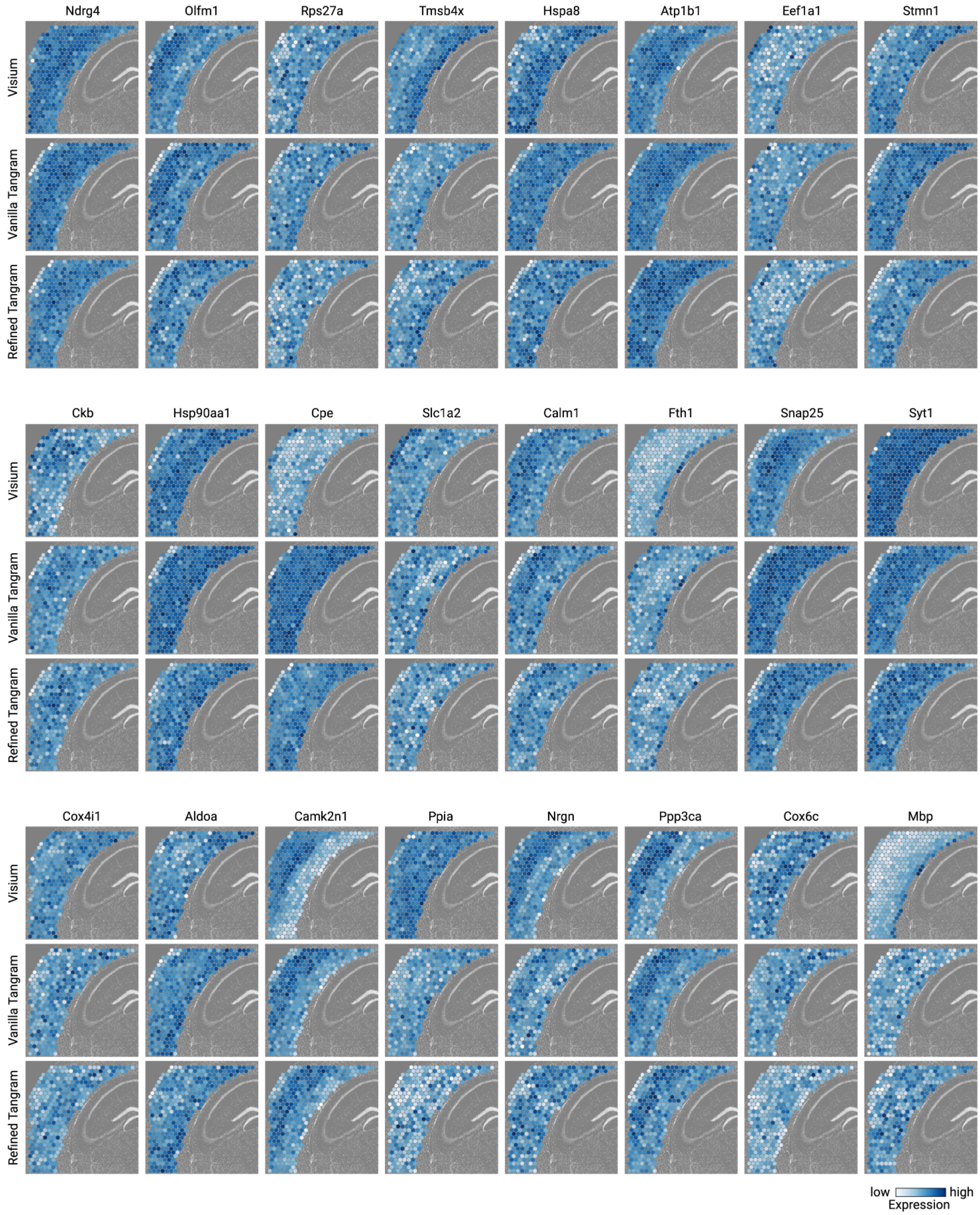

Fig. 3: **Comparison of predicted gene expressions on real-world mouse cortex scRNA-seq and Visium data.** We refined the cell mapping algorithm of Tangram by adding gene and cell selection, neighborhood incorporation, and regularization. The vanilla Tangram (middle rows) and the refined Tangram (lower rows) were applied to map the scRNA-seq expression data to spatial data. The spatial gene expression is then predicted by multiplying those cell mappings with the single-cell gene expression. The figure compares the normalized predicted gene expressions of the 27 spatially variable genes (predicted with SpatialDE2 on the Visium dataset) with the largest read counts to the normalized Visium ground truth (upper rows).

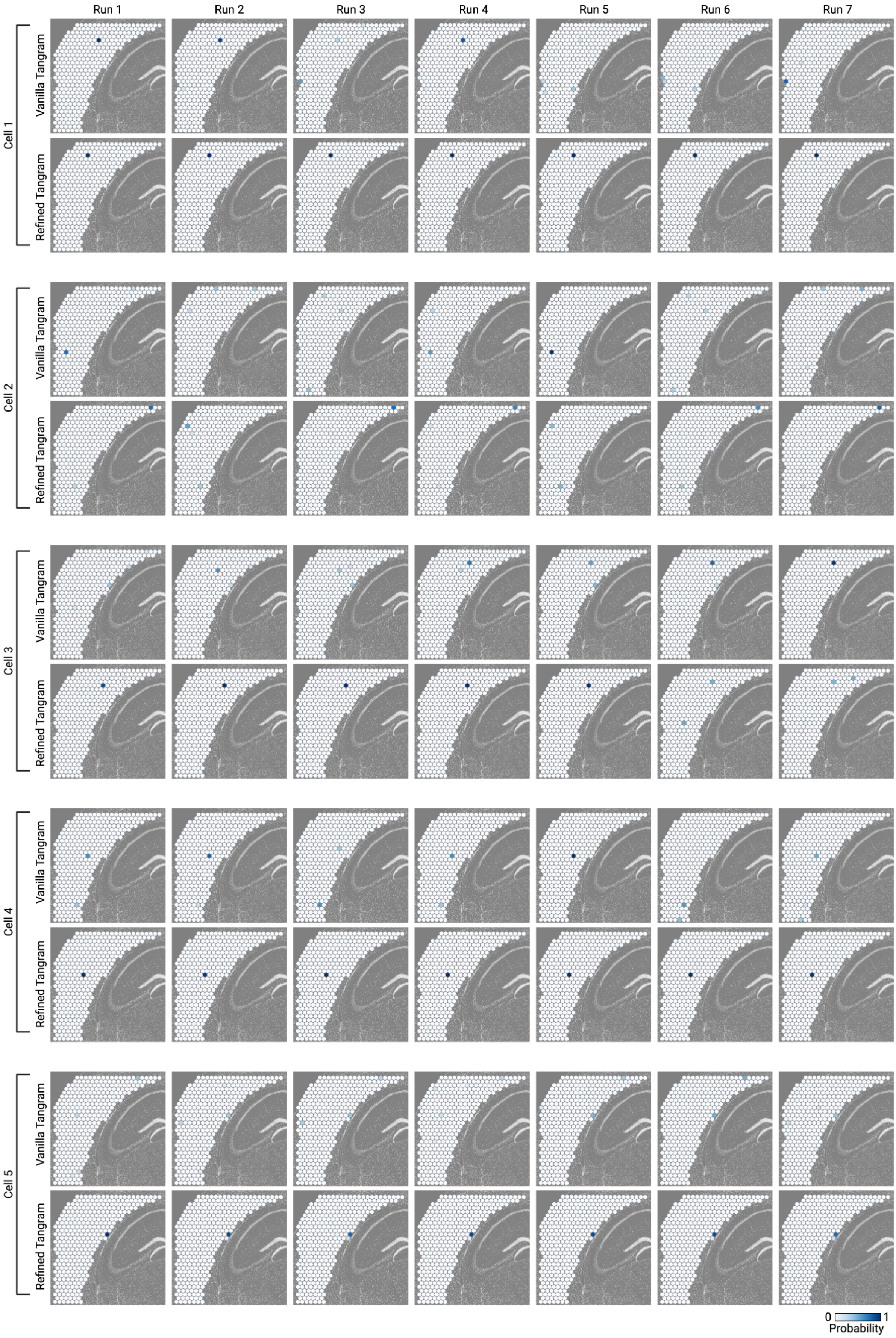

Fig. 4: **Comparison of cell mappings on real-world mouse cortex scRNA-seq and Visium data.** We refined the cell mapping algorithm of Tangram by adding gene and cell selection, neighborhood incorporation, and regularization. The vanilla Tangram (upper rows) and the refined Tangram (lower rows) were repeatedly applied to map the scRNA-seq expression data to spatial data. The figure compares selected cell mappings over 7 runs. It demonstrates, that Tangram refinements improve consistency, agreement, and certainty.

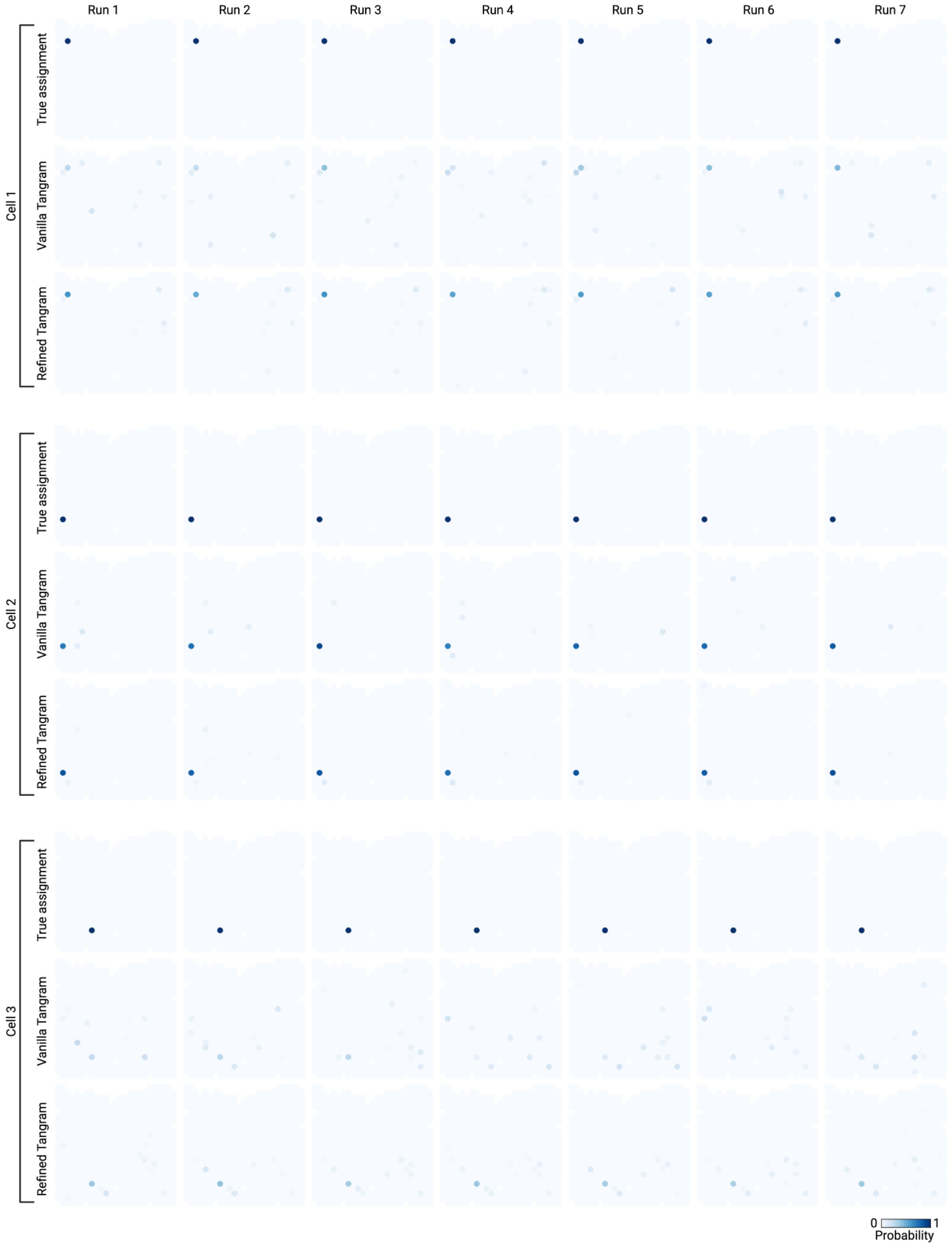

Fig. 5: **Comparison of cell mappings on simulated mouse hypothalamus scRNA-seq and spatial data.** We refined the cell mapping algorithm of Tangram by adding gene and cell selection, neighborhood incorporation, and regularization. The vanilla Tangram (middle rows) and the refined Tangram (lower rows) were repeatedly applied to map the scRNA-seq expression data to spatial data. The figure compares selected cell mappings over 7 runs and to the ground truth (upper row). It demonstrates, that Tangram refinements improve consistency, certainty, and correctness.

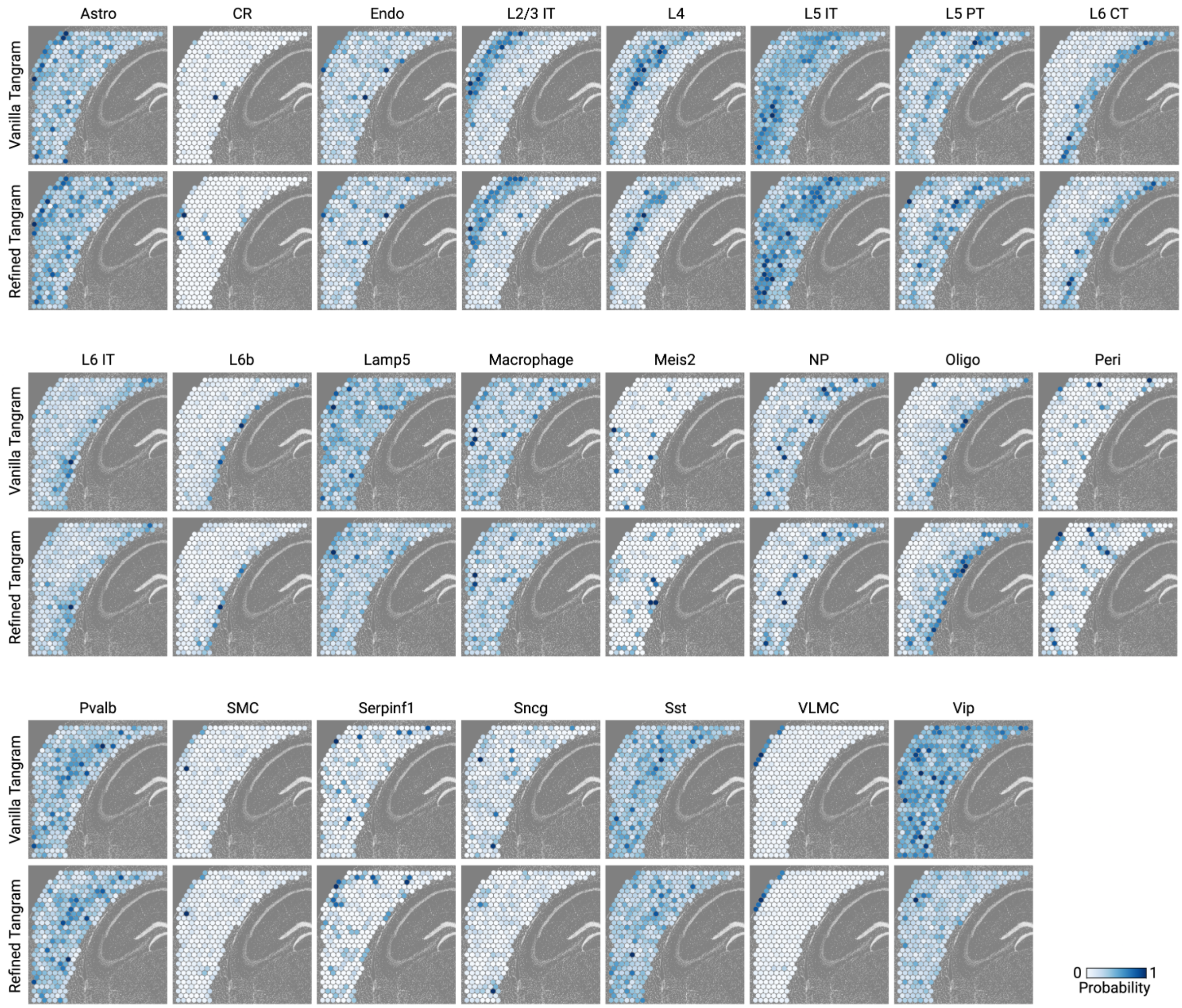

Fig. 6: **Comparison of cell type mappings on real-world mouse cortex scRNA-seq and Visium data.** We refined the cell mapping algorithm of Tangram by adding gene and cell selection, neighborhood incorporation, and regularization. The predicted cell type mappings using vanilla Tangram (upper rows) are compared to the ones using refined Tangram (lower rows). Cell type probabilities are computed from the cell mappings by summing up cell mapping probabilities each cell type and normalization by the maximum value per type.

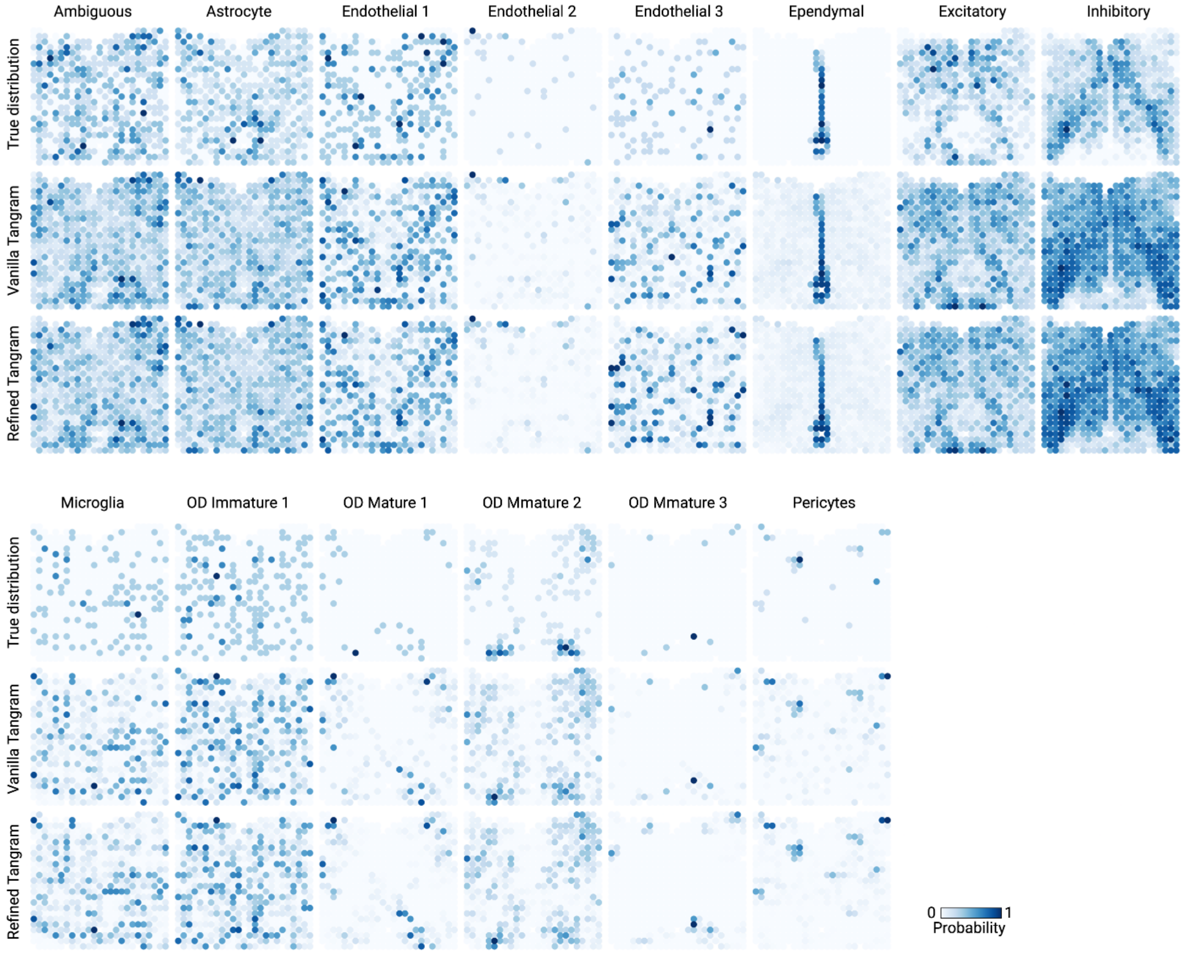

Fig. 7: **Comparison of cell type mappings on simulated mouse hypothalamus scRNA-seq and spatial data.** We refined the cell mapping algorithm of Tangram by adding gene and cell selection, neighborhood incorporation, and regularization. True spatial distributions (upper rows) are compared to the predicted cell type mappings using vanilla Tangram (middle rows) and the refined Tangram (lower rows). Types with less than 10 cells are excluded. Cell type probabilities are computed from the cell mappings by summing up cell mapping probabilities each cell type and normalization by the maximum value per type.

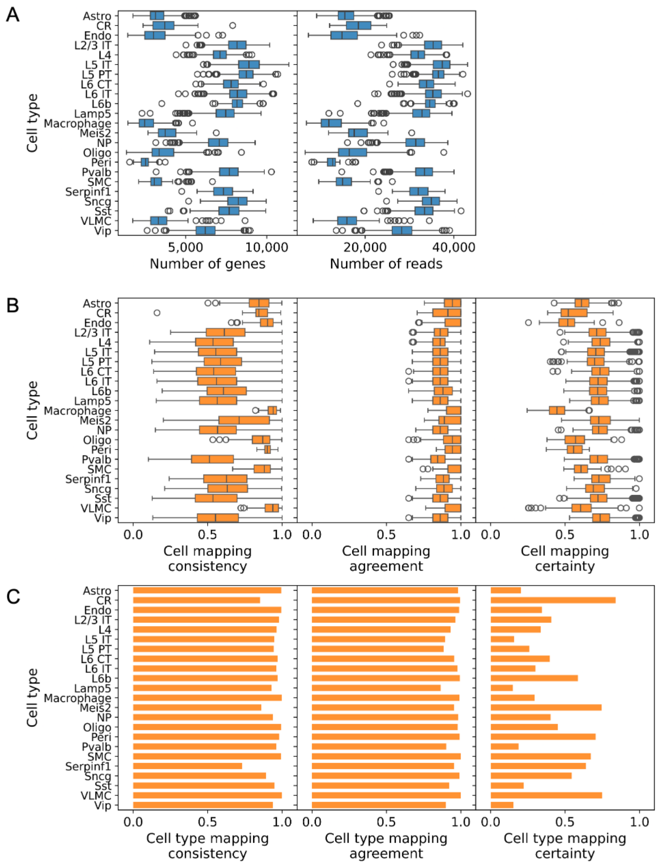

Fig. 8: **Cell and cell type mapping per cell type.** Mouse brain scRNA-seq data was mapped to spatial data using Tangram and evaluated. The number of genes and reads in the scRNA-seq (A), cell mapping metrics (B) and cell type mapping metrics (C) over 10 Tangram runs are shown per cell type.

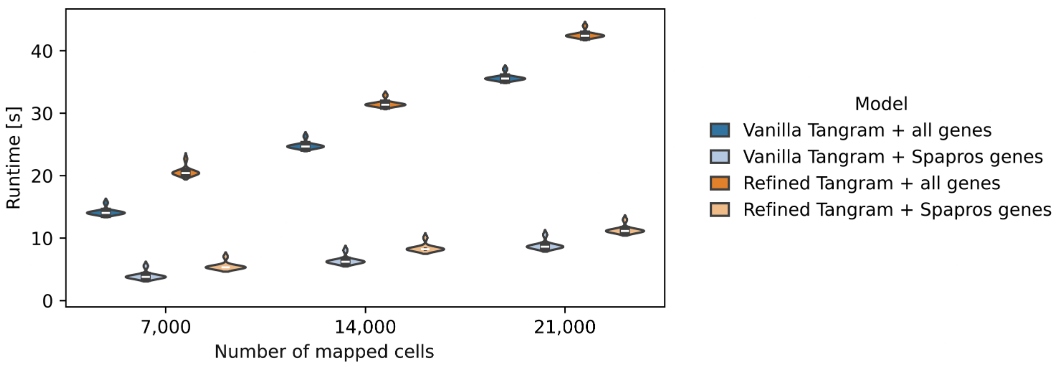

Fig. 9: **Runtimes analysis on real-world mouse cortex scRNA-seq and Visium data.** We refined the cell mapping alogrithm of Tangram by adding cell and gene selection, neighborhood incorporation, and regularization. The vanilla Tangram and the refined Tangram was trained on either all 14,785 genes or the 1,763 Spapros genes on two Intel(R) Xeon(R) Silver 4114 CPUs and one NVIDIA A40 64-Core GPU. The figure shows the runtimes over 10 runs per configuration stratified by different numbers of mapped cells. While our refinements introduce a slight computational overhead, the results demonstrate that gene set selection has the most impact on runtime performance.
